# 

**Published:** 1861-03

**Authors:** 


### CONTENTS FOR MARCH, 1861.

### ORIGINAL COMMUNICATIONS.

Nature and Art. Their relative influence in the management of Diseases. Are they antagonistic or co-operative? An Essay read to the Chicago Medical Society, Oct. 19th, 1860. By N. S. DAVIS, M. D. .... 129

Valedictory Address to the Graduates of Medical Department of Lind University, for the Session of 1860-61. By M. K. TAYLOR, M. D., Professor of Pathology and Public Hygiene .... 139

Notes on Surgical Cases. By E. ANDREWS, M. D. .... 148

Diphtheria, its Nature and Treatment. By C. W. DAVIS, M. D. .... 150

Clinic of Prof. DAVIS in the Mercy Hospital. Reported by F. S. C. Grayston, Member of Senior Class Med. Department of Lind University .... 153

Chicago Academy of Medical Science .... 162

Abstract of the Proceedings of the Will Co. Medical Society .... 164

Chicago Medical Society .... 165

### SELECTIONS.

Clinical Remarks on Sciatic Neuralgia, with a Case. By JOSEPH PANCOAST, M. D. .... 166

Clinical Remarks on Acute Articular Rheumatism. By FRANCIS G. SMITH, M. D. .... 170

Prof. Millar on Tobacco .... 174

On the Action of Iodide of Iron upon Phthisis .... 178

### BOOK AND PAMPHLET NOTICES.

An Elementary Treatise on Human Anatomy. By JOSEPH LEIDY, M. D. .... 180

Electro-Physiology and Electro-Therapeutics. By ALFRED C. GRANETT, M. D. .... 181

A Practical Treatise on Phthisis Pulmonalis. By L. M. LAWSON, M. D. .... 181

Lives of Eminent American Physicians and Surgeons of the Nineteenth Century. By SAMUEL D. GROSS, M. D. .... 182

Theory and Practice of the Movement-Cure. By CHARLES FAYETTE TAYLOR, M. D. .... 183

Diphtheria, Its Nature and Treatment. By DANIEL DENISON SLADE, M. D. .... 183

A Treatise on Fever. By REZIN THOMPSON, M. D. .... 184

### EDITORIAL.

Illinois State Medical Society .... 185

Medical College Commencements in this City .... 185

Medical Department of Lind University .... 186

Berkshire Medical Journal .... 187

Change of Name .... 187

New York Ophthalmic School .... 187

Summer Course of Medical Instruction .... 188

Homœopathy and the Michigan University .... 188

The New Sydenham Society .... 189

Obituary .... 191

